# Complete remission of recurrent gastric cancer in a young female patient through CLDN18.2-targeted therapy (LM302) and metastatic ovarian tumor resection: a case report of refractory disease overcoming chemotherapy and immunotherapy resistance

**DOI:** 10.3389/fonc.2025.1631062

**Published:** 2025-08-18

**Authors:** Muyang Chen, Tongshan Wang, Anpeng Wang, Hao Zhang

**Affiliations:** 1 School of Pediatrics, Nanjing Medical University, Nanjing, Jiangsu, China; 2 Gastric Cancer Center, Department of Oncology, the First Affiliated Hospital of Nanjing Medical University, Nanjing, China; 3 Department of Oncology, First Affiliated Hospital of Nanjing Medical University, Nanjing, Jiangsu, China

**Keywords:** gastric cancer, CLDN18.2-targeted therapy, LM302, ovarian metastasis, precision medicine

## Abstract

Young patients with recurrent, metastatic gastric cancer (GC) resistant to chemotherapy and immunotherapy have poor outcomes and limited treatment options. CLDN18.2 has emerged as a promising target in GC. We report the case of a 35-year-old female who experienced recurrence with bilateral ovarian and lymph node metastases 16 months after radical gastrectomy and adjuvant chemotherapy. Multiple therapies failed, and CLDN18.2 expression was suspected. She enrolled in a Phase I/II trial of LM302, a CLDN18.2-targeted antibody-drug conjugate, receiving eight cycles. Imaging showed significant regression of lymph node lesions and stable ovarian metastases. In April 2024, she underwent laparoscopic resection of the ovarian tumors, with pathology confirming no residual disease. As of March 2025, she remains disease-free with excellent performance status. This case illustrates that CLDN18.2-targeted therapy combined with surgery may offer curative potential in refractory GC and highlights the importance of biomarker-driven precision medicine.

## Introduction

Gastric cancer (GC) remains one of the most prevalent and fatal malignancies worldwide, ranking fifth in incidence and third in cancer-related mortality. Prognosis is closely associated with the disease stage at diagnosis. Although radical surgery combined with perioperative chemotherapy improves survival in patients with locally advanced GC, postoperative recurrence rates remain high, ranging from 30% to 50%. Recurrence is often accompanied by distant metastases (e.g., ovaries, lymph nodes), resulting in a dismal five-year survival rate of less than 10% (1). Notably, younger patients with GC (<40 years) exhibit more aggressive clinical characteristics and a significantly higher risk of recurrence compared to older individuals. Additionally, resistance to conventional chemotherapy and immunotherapy is more pronounced in this cohort. However, overall survival (OS) differences between younger and older patients remain controversial (2, 3). Regardless, there is an urgent need to explore biomarker-driven, precision therapeutic strategies.

In recent years, Claudin 18.2 (CLDN18.2) has emerged as a highly selective tumor-associated antigen. This protein is specifically overexpressed in 60%-80% of gastric adenocarcinomas while being restricted to differentiated epithelial cells of the gastric mucosa in normal tissues, making it an attractive therapeutic target. Reports indicate that CLDN18.2 positivity in refractory advanced GC reaches approximately 40% or higher (4–6). Targeted therapies against CLDN18.2, including monoclonal antibodies and antibody-drug conjugates (ADCs), have demonstrated remarkable antitumor efficacy in clinical trials.

Among these agents, LM302, a novel CLDN18.2-targeted ADC, delivers a cytotoxic payload to tumor cells via monoclonal antibody-mediated targeting. Preliminary results from its Phase I/II clinical trial (NCT05161390) demonstrated promising antitumor activity in heavily pretreated patients with gastric or gastroesophageal junction (GEJ) cancer. Among 36 evaluable patients who had received at least two prior lines of therapy, 11 achieved partial response (PR) and 16 had stable disease (SD). The objective response rate (ORR) was 30.6% (11/36), and the disease control rate (DCR) reached 75.0% (27/36). The median progression-free survival (PFS) was 7.16 months (95% CI: 2.72-NA). The median OS was not yet reached, with a six-month OS rate of 95.0% (7). However, clinical data regarding its combination with surgical resection to achieve curative outcomes in metastatic settings remain limited.

Multidisciplinary treatment approaches are increasingly recognized in the management of advanced GC. Notably, a strategy involving targeted therapy-induced tumor burden reduction followed by sequential surgical resection of residual lesions may offer a curative potential for selected patients with oligometastatic disease (8). However, patients resistant to conventional chemotherapy and immunotherapy often lack effective systemic treatment options and are typically excluded from such aggressive interventions.

Here, we report a case of a 35-year-old female with metastatic GC who exhibited resistance to multiple lines of therapy, including chemotherapy and immunotherapy. Through the integration of CLDN18.2-targeted therapy (LM302) with metastasectomy, she achieved long-term disease-free survival. This case provides novel insights into overcoming therapeutic resistance in young patients with refractory gastric cancer and highlights the potential of biomarker-driven precision medicine combined with surgical intervention.

## Case report

A 36-year-old female patient presented with upper abdominal discomfort in August 2021. Gastroscopy revealed multiple ulcers with hemorrhage on the greater curvature of the gastric body, highly suggestive of malignancy. In September 2021, she underwent laparoscopic radical total gastrectomy for gastric cancer at Nanjing First Hospital. Postoperative pathology confirmed moderately differentiated tubular adenocarcinoma of the greater curvature of the gastric body (pT3N3bMx) with extensive lymph node metastases (10/18 on the lesser curvature and 9/16 on the greater curvature) and vascular invasion.

Adjuvant chemotherapy was initiated in November 2021 with oxaliplatin plus tegafur-gimeracil-oteracil (S-1). Due to gastrointestinal toxicity, the regimen was adjusted to oxaliplatin plus capecitabine. Further modifications were necessary due to bone marrow suppression and febrile episodes, leading to a regimen of docetaxel plus capecitabine, which was ultimately maintained as capecitabine monotherapy until September 2022. In February 2023, PET-CT evaluation indicated disease progression (PD) with bilateral ovarian metastases (increased FDG uptake) and extensive lymph node metastases in the left supraclavicular and left pulmonary regions. Second-line therapy was initiated, comprising camrelizumab, paclitaxel micelles, and lenvatinib, combined with hyperthermia and ultrasonic knife therapy. However, the patient experienced severe adverse effects, including high fever and vomiting, prompting a regimen adjustment to sintilimab, paclitaxel micelles, and lenvatinib, which remained ineffective.

In July 2023, a multidisciplinary discussion, supported by literature and clinical experience, strongly suggested high CLDN18.2 expression in the tumor, which was confirmed by immunohistochemical testing of the tumor tissue removed during the first operation at another hospital (but unfortunately we were unable to find the original data of the patient’s immunohistochemical testing at another hospital),making the patient eligible for enrollment in the Phase I/II clinical trial of LM302, a CLDN18.2-targeted antibody-drug conjugate (ADC). On August 4, 2023, the patient, who had previously exhibited sinus arrhythmia, demonstrated normalization of heart rhythm, signed informed consent, and was enrolled in the study. She commenced LM302 therapy (2.4 mg/kg Q3W). Pre-treatment evaluation showed an ECOG score of 0, stable ovarian and lymph node metastases, and no significant laboratory abnormalities (See **Supplementary Figures S1–3** for details).

Following LM302 initiation, the patient experienced nausea, which was classified as CTCAE grade 1 until January 12, 2024, when it progressed to CTCAE grade 2. The nausea was attributed to LM302, but no dose adjustments were made. Instead, prophylactic intravenous lansoprazole (30 mg) was administered to protect the gastric mucosa, alongside oral palonosetron hydrochloride for antiemesis and granulocyte colony-stimulating factor (G-CSF) to prevent leukopenia and neutropenia. Under G-CSF stimulation, the patient’s white blood cell count transiently increased to 32.39 × 10^9^/L, with a neutrophil percentage of 90.30%, while red blood cell count and hemoglobin levels slightly declined (See **Supplementary Figures S4, 5** for details). These findings indirectly indicated that LM302 did not induce significant bone marrow suppression.

On January 25, 2024, CT scans revealed substantial absorption of subpleural patchy opacities in the upper lobe of the left lung, while the right lung remained largely unchanged. Bilateral ovarian metastatic lesions showed partial regression. However, by April 18, 2024, CT scans detected slight enlargement of the bilateral ovarian metastatic lesions, whereas multiple small pulmonary nodules and mesenteric lymph nodes adjacent to the abdominal aorta remained stable (See **Supplementary Figures S6, 7** for details). At this point, an extraordinary observation was made: with the exception of a few of slightly enlarged ovarian metastatic lesions, all other metastatic lesions had regressed, and the involved lymph nodes had resolved. In other words, according to the tumor immune surveillance threshold hypothesis recognized in the academic literature (21), the tumor burden at all sites other than the ovary may have declined below the threshold required for immune-mediated clearance, thereby allowing the host immune system to eliminate the remaining cancer cells. So in early May 2024, the patient underwent bilateral oophorectomy. Approximately one week postoperatively, she developed acute erythematous papules with pruritus on both hands. Dermatology specialists managed the condition with an intravenous bolus of dexamethasone sodium phosphate (5 mg), followed by oral levocetirizine hydrochloride, triclosan cream, and compounded lidocaine cream, leading to resolution within a week (See **Supplementary Figures S8, 9** for details). Postoperative immunohistochemistry of the resected ovarian tumors confirmed PD-L1 negativity (See **Supplementary Figures S10** for details). Enhanced CT images of the patient’s metastatic lesions obtained prior to LM302 treatment (July 24, 2023), as well as those acquired before bilateral oophorectomy following LM302 treatment (January 25 and April 18, 2024), are presented in **Supplementary Figures S11–17**. Corresponding explanatory notes have been added for reference.

Following oophorectomy, the patient discontinued all anticancer treatments and has since undergone regular follow-up. To date, she remains in good health, with no signs of recurrence and an ECOG score maintained at 0. All medical records and examination results are provided in the **Supplementary Materials**.

## Discussion

This case report presents the treatment process of a young female patient with refractory gastric cancer who achieved complete remission through CLDN18.2-targeted therapy with LM302 combined with metastasectomy. The discussion below integrates the latest research advances from the perspectives of molecular mechanisms, clinical strategies, and patient-specific factors.

CLDN18.2, a member of the tight junction protein family, is highly expressed in gastric cancer and plays a crucial role in tumor proliferation, invasion, and metastasis (9). LM302, a CLDN18.2-targeted antibody-drug conjugate (ADC), selectively delivers cytotoxic payloads (such as monomethyl auristatin E, MMAE) to CLDN18.2-positive tumor cells via antibody-mediated endocytosis while minimizing toxicity to normal tissues (10). In this case, despite the absence of standardized CLDN18.2 testing results (due to early limitations in medical conditions), the patient exhibited a significant response to LM302, with complete regression of lymph node metastases. This aligns with recent findings from a Phase III clinical trial of zolbetuximab, a CLDN18.2 inhibitor, combined with mFOLFOX6 versus standard chemotherapy (mFOLFOX6) in CLDN18.2-positive, HER2-negative gastric and gastroesophageal junction (GEJ) adenocarcinoma, where efficacy correlated with CLDN18.2 expression levels (11). Additionally, the patient’s ovarian metastases only achieved stable disease (SD) following LM302 treatment, suggesting potential tumor heterogeneity, with CLDN18.2 expression or drug penetration in metastatic lesions being lower than in the primary tumor. Similar phenomena have been observed in breast cancer mouse models, where disseminated tumors exhibit significant variability in vascular density (CD31) and tumor marker expression (mKate, Her2/neu), while primary tumors show relatively less heterogeneity (12). This highlights the need for multi-regional biopsies or liquid biopsies for further validation.

Our clinical observations suggest that immunotherapy represents a critical—if not the only—therapeutic modality capable of enabling surgical intervention or achieving remission in patients with advanced, unresectable malignancies. This phenomenon may be explained by the tumor immune surveillance threshold theory (21), which posits that immunotherapy can, under appropriate conditions, elevate the host’s immune surveillance threshold—that is, the maximal tumor burden the immune system can recognize and eliminate—thereby facilitating complete tumor eradication. Notably, the patient was resistant to PD-1 inhibitors (camrelizumab and sintilimab), and postoperative immunohistochemistry of ovarian metastases confirmed PD-L1 negativity, suggesting that immune therapy resistance might be linked to an immunosuppressive tumor microenvironment (TME). Recent studies indicate that CLDN18.2-targeted therapy can induce immunogenic cell death (ICD), thereby activating T-cell responses and converting “cold” tumors into “hot” tumors, demonstrating synergy with PD-1/PD-L1 inhibitors (13). Moreover, a bispecific antibody targeting both CLDN18.2 and PD-L1 (Q-1802) is currently under investigation (14). Notably, the patient in this case did not receive anti-PD-1/PD-L1 immune checkpoint inhibitors during treatment with LM302, highlighting a potential avenue for future investigation into combinatorial therapeutic strategies, particularly for patients with refractory disease. In future cases, it may be valuable to monitor dynamic changes in PD-1/PD-L1 expression during treatment with CLDN18.2-targeted immunotherapies. Should a transition from negative to positive expression be observed, the addition of an anti-PD-1/PD-L1 agent to establish a dual-antibody therapeutic regimen could potentially enhance clinical efficacy. Another study demonstrated that gastric cancer patients with high CLDN18.2 expression (≥40%) exhibited a more complex immune microenvironment. Specifically, the proportions of CD8^+^PD-1^-^, CD8^+^LAG-3^-^, and CD8^+^TIM-3^-^ T cells were significantly higher in the CLDN18.2-positive group compared to the negative control group (0.039 vs. 0.026, P = 0.009; 0.050 vs. 0.035, P = 0.024; 0.045 vs. 0.032, P = 0.038, respectively). In addition, neutrophil infiltration (CD66b^+^) was significantly greater in the CLDN18.2-positive group than in the negative group (0.081 vs. 0.055, P = 0.031). Conversely, the proportions of M1 macrophages (CD68^+^CD163^-^HLA-DR^+^), M2 macrophages (CD68^+^CD163^+^HLA-DR^-^), and B cells (CD20^+^) were comparable between the two groups. These findings are consistent with our earlier observations that CLDN18.2 positivity may attenuate the efficacy of PD-1/PD-L1 inhibitors, and further support the rationale for considering CLDN18.2-targeted CAR-T cell therapy as a promising treatment strategy for this subset of gastric cancer patients (22). Another study conducted by the same corresponding author was a phase I clinical trial evaluating Claudin18.2-specific CAR-T cell therapy in patients with gastrointestinal cancers, which yielded promising results. Among the 98 enrolled patients, the overall response rate (ORR) and disease control rate (DCR) were 38.8% and 91.8%, respectively. The median progression-free survival (PFS) was 4.4 months (95% CI: 3.7–6.6), and the median overall survival (OS) was 8.8 months (95% CI: 7.1–10.2). These findings indirectly support the hypothesis that CLDN18.2-targeted CAR-T cell therapy may be an effective treatment option in this patient population (23).

For oligometastatic gastric cancer, the feasibility of systemic therapy combined with radical local surgery is gaining increasing attention. In this case, LM302 significantly reduced lymph node metastases, whereas ovarian metastases remained stable, ultimately necessitating surgical resection to achieve a tumor-free status. This outcome underscores the efficacy of a strategy involving initial systemic therapy followed by surgery, regardless of initial tumor resectability. Notably, a study comparing perioperative SOX chemotherapy with adjuvant CAPOX chemotherapy in resectable gastric cancer demonstrated superior three-year disease-free survival (59.4% vs. 51.1%, P = 0.028) (15). Another small-scale Phase II study investigated trastuzumab combined with DCS chemotherapy in 16 patients with HER2-positive unresectable metastatic gastric cancer, reporting an objective response rate (ORR) of 93.8% (15/16) and an R0 resection rate of 56.3% (9/16) (16).

However, not all patients are suitable for such aggressive interventions. Based on this case, we propose a “metastatic dynamics assessment model” to identify candidates for complete metastasectomy. The following criteria should be met: (1) systemic therapy is effective, and metastatic lesions are limited, well-defined, and manageable (including lymph node involvement); (2) no new metastases appear over a sustained period; and (3) the patient maintains a good performance status (ECOG ≤1). The present case met these criteria, suggesting the model’s potential clinical applicability. Further refinement and validation of this model in clinical practice will be essential.

Young patients with gastric cancer (<40 years) often harbor unique molecular characteristics, including a higher prevalence of genomic instability (e.g., TP53 mutations and ARID1A loss) and epigenetic abnormalities (17). The rapid progression and multi-line treatment resistance observed in this case may be linked to the highly aggressive molecular phenotype of the tumor. Recent pooled analyses suggest that CLDN18.2 positivity is more frequent in younger gastric cancer patients than in older counterparts (18, 19), providing a rationale for the preferential use of CLDN18.2-targeted therapies in this population. Furthermore, younger patients generally exhibit better treatment tolerance, making them ideal candidates for intensive combination strategies (e.g., ADC therapy plus surgery), as evidenced by this patient’s successful completion of eight cycles of LM302 therapy followed by surgery.

This study has several limitations: (1) The original immunohistochemistry report confirming CLDN18.2 positivity from the referring hospital was unavailable, so it is impossible to explain the specific expression level of CLDN18.2, meanwhile, the PD-1/PD-L1 status of the initial endoscopic biopsy remains uncertain(However, given the PD-L1 negativity observed in the tumor tissue resected during the final oophorectomy, it is likely that the primary lesion was also PD-1/PD-L1 negative). This may limit the evidentiary strength and reduce the overall persuasiveness of the findings; (2) As a single case report, chance findings cannot be ruled out, and prospective cohort studies are needed for validation; and (3) the long-term toxicity of LM302, including potential neurotoxicity and cardiotoxicity, remains unclear. Future research should prioritize the optimization of predictive biomarkers through the development of circulating tumor DNA (ctDNA)-based strategies for monitoring molecular response, as well as the relative abundance and activity of tumor cells in CLDN18.2-positive gastric cancer. Such approaches may improve the ability to assess recurrence risk and guide clinical decision-making regarding the timing of therapeutic interventions and surgical resection (20). Unfortunately, during the treatment period of the patient described in this case report, circulating tumor DNA (ctDNA) detection technology was not yet widely accessible in clinical practice. At that time, the technique remained relatively immature, was not covered by China’s national medical insurance system, and required full out-of-pocket payment, rendering it infeasible for continuous monitoring throughout the patient’s treatment course. Moving forward, we aim to facilitate broader access to ctDNA-based monitoring for similar patients, as it holds great potential in guiding treatment decisions and evaluating therapeutic efficacy in real time. Additionally, while the potential synergy between CLDN18.2-targeted therapy and PD-1/PD-L1 inhibitors has been discussed, we hypothesize that combining CLDN18.2-targeted therapy with anti-angiogenic agents (e.g., lenvatinib and anlotinib) may enhance tumor penetration and anti-tumor efficacy, offering a novel option for patients with multi-line resistance.

## Conclusion

This case report provides the first clinical evidence that CLDN18.2-targeted therapy with LM302 combined with metastasectomy can be effective in young patients with refractory gastric cancer. The success of this approach underscores the importance of biomarker-driven treatment decisions and multidisciplinary collaboration. With the standardization of CLDN18.2 testing and the development of novel combination strategies, individualized treatment approaches have the potential to reshape the therapeutic landscape of advanced gastric cancer.

## Data Availability

The original contributions presented in the study are included in the article/**Supplementary Material**. Further inquiries can be directed to the corresponding author.
