# Supplementary material for: Complete remission of recurrent gastric cancer in a young female patient through CLDN18.2-targeted therapy (LM302) and metastatic ovarian tumor resection: a case report of refractory disease overcoming chemotherapy and immunotherapy resistance
Source: Front Oncol. 2025 Aug 18;15:1631062. doi: 10.3389/fonc.2025.1631062 (PMC12400029; doi:10.3389/fonc.2025.1631062)
Supplement: Supplementary file 1 [file SupplementaryFile1.docx]

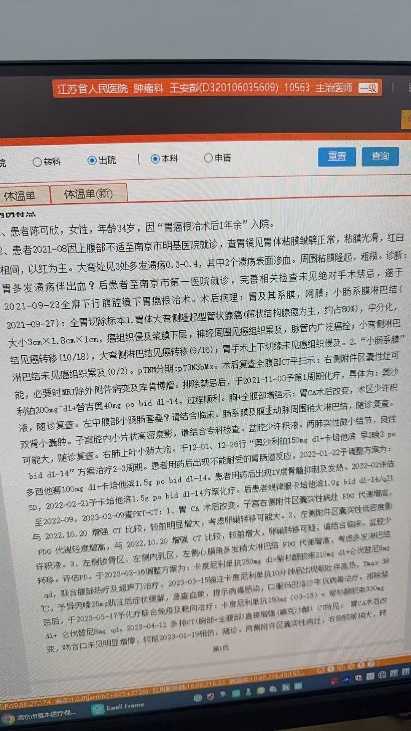


他

Figure 1 Medical history before LM302 administration (1)


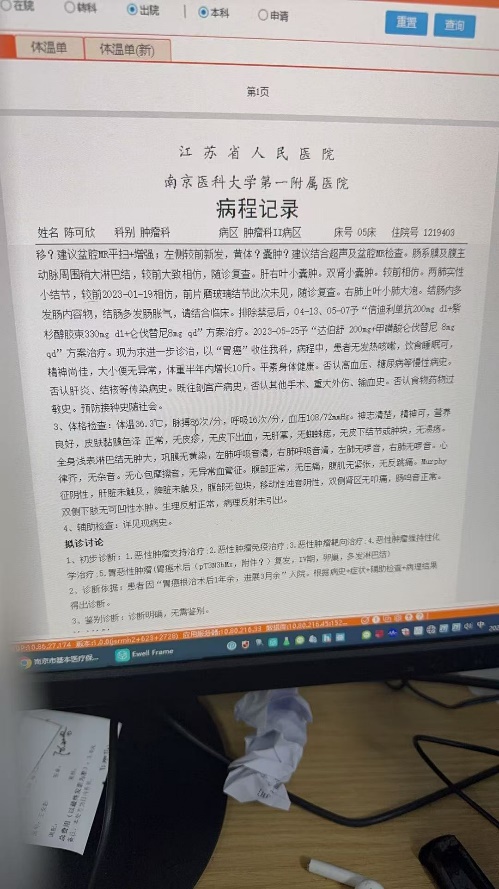


Figure 2 Medical history before LM302 administration (2)


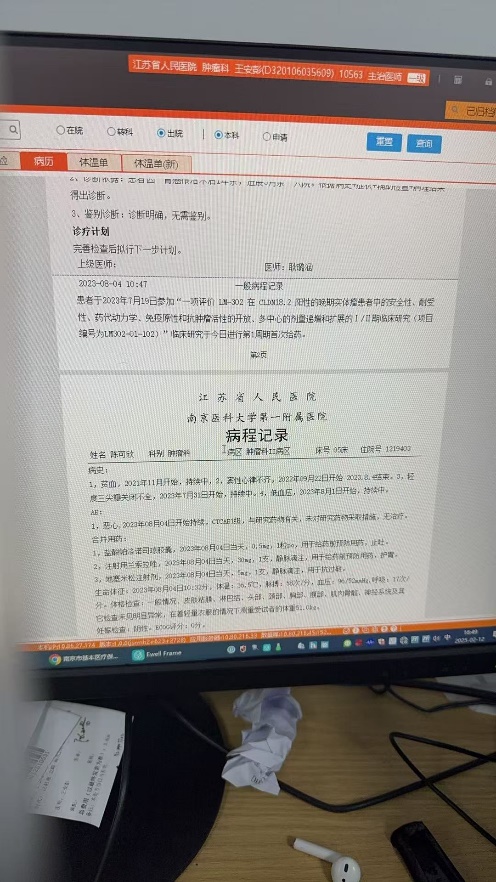


Figure 3 Medical history before LM302 administration (3)

Description of Figures 1 to 3 The patient, a 34-year-old female, was admitted with a history of intermittent cough and expectoration for over a year, which had worsened over the past two months. She had a known diagnosis of chronic bronchiectasis with recurrent infections, confirmed by chest CT showing signs of bronchiectasis and pulmonary infection. Upon admission, she presented with productive cough, purulent sputum, and occasional hemoptysis, accompanied by elevated inflammatory markers. Treatment included intravenous antibiotics (such as cefoperazone/sulbactam and moxifloxacin), antitussives, oxygen therapy as needed, and supportive care. Sputum culture and drug sensitivity tests were conducted to guide targeted antimicrobial therapy. Chest physiotherapy and nebulized treatments were administered to promote airway clearance. The patient's clinical condition gradually improved, with a significant reduction in cough and sputum production. At discharge, she was afebrile and clinically stable. The discharge plan included oral antibiotics, ongoing pulmonary rehabilitation, avoidance of respiratory infections, and follow-up imaging (chest CT) in 1–2 months to monitor for recurrence or complications.


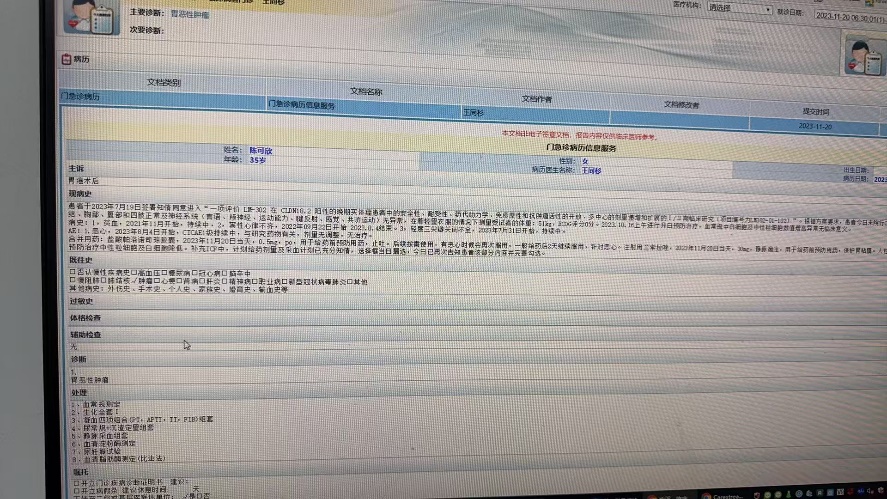


Figure 4 Medical history after LM302 administration (1) After the start of LM302 treatment, the patient developed drug-related nausea symptoms, which were CTCAE grade 1 from the start of treatment to January 12, 2024. It progressed to CTCAE grade 2 on January 12, 2024, which was related to LM302. No dose adjustment was required, but 30 mg of lansoprazole for injection was given intravenously before administration to protect the gastric mucosa, palonosetron hydrochloride capsules were taken orally to prevent vomiting, and human granulocyte colony-stimulating factor was taken to prevent a decrease in total white blood cell count and neutrophil count.


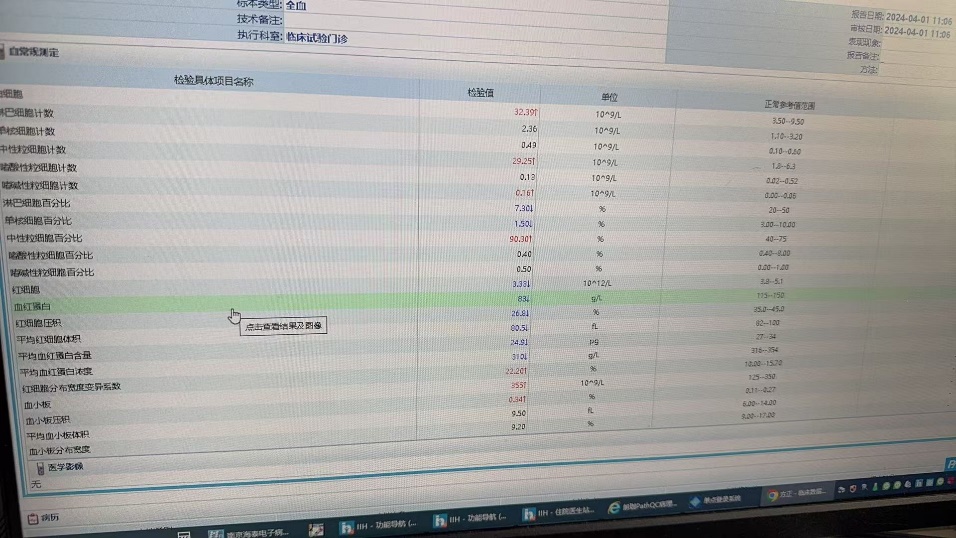


Figure 5 Medical history after LM302 administration (2)

Under the action of the stimulating factor, the patient's total white blood cell count once reached 32.39*10^9, and the percentage of neutrophils reached 90.30%. At the same time, the patient's red blood cells and hemoglobin decreased slightly.


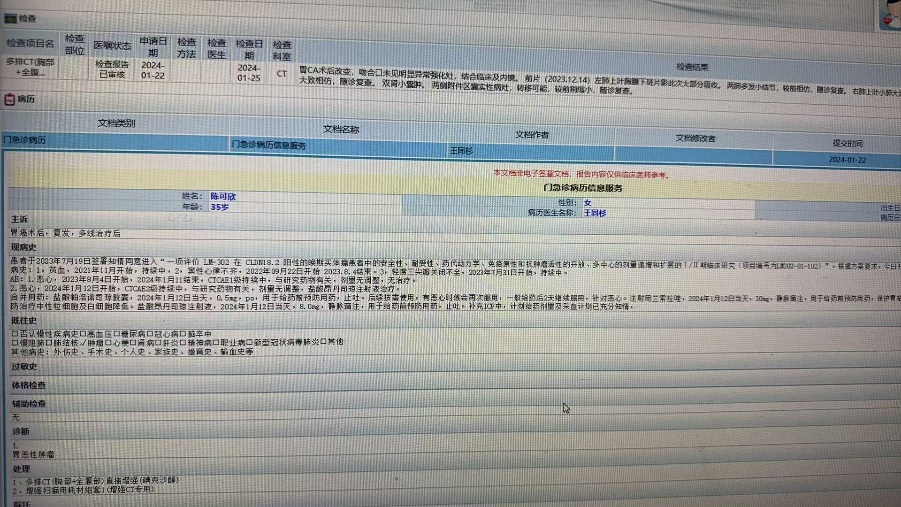


Figure 6 2024.1.25 CT examination record showed that most of the subpleural patchy shadows in the left upper lobe of the lung were absorbed, and the right side remained roughly unchanged. The cystic and solid metastatic lesions in the adnexal areas on both sides were reduced.


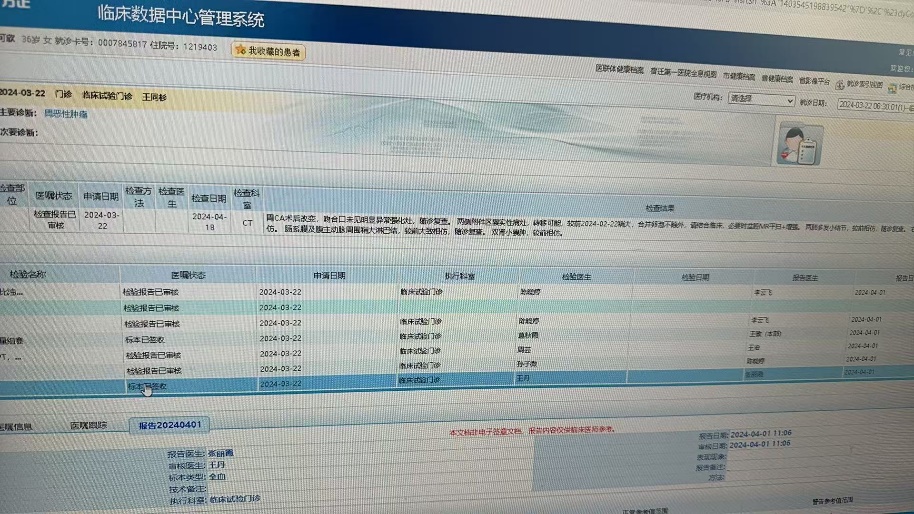


Figure 7 2024.4.18 CT examination record

CT scan on April 18, 2024 revealed that the cystic and solid metastatic lesions in the adnexal areas on both sides had increased in size, while the multiple small nodules in both lungs and the slightly larger lymph nodes around the mesenteric abdominal aorta remained basically unchanged.


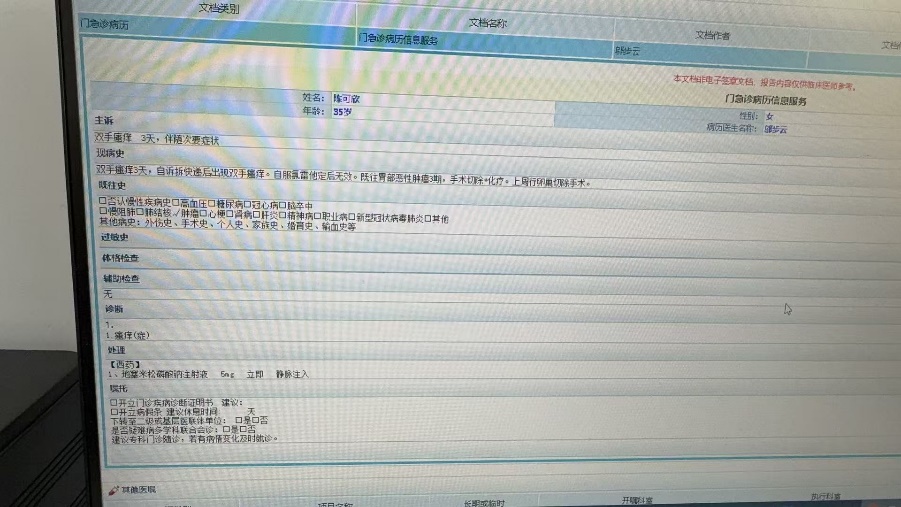


Figure 8 Dermatology medical records (1)


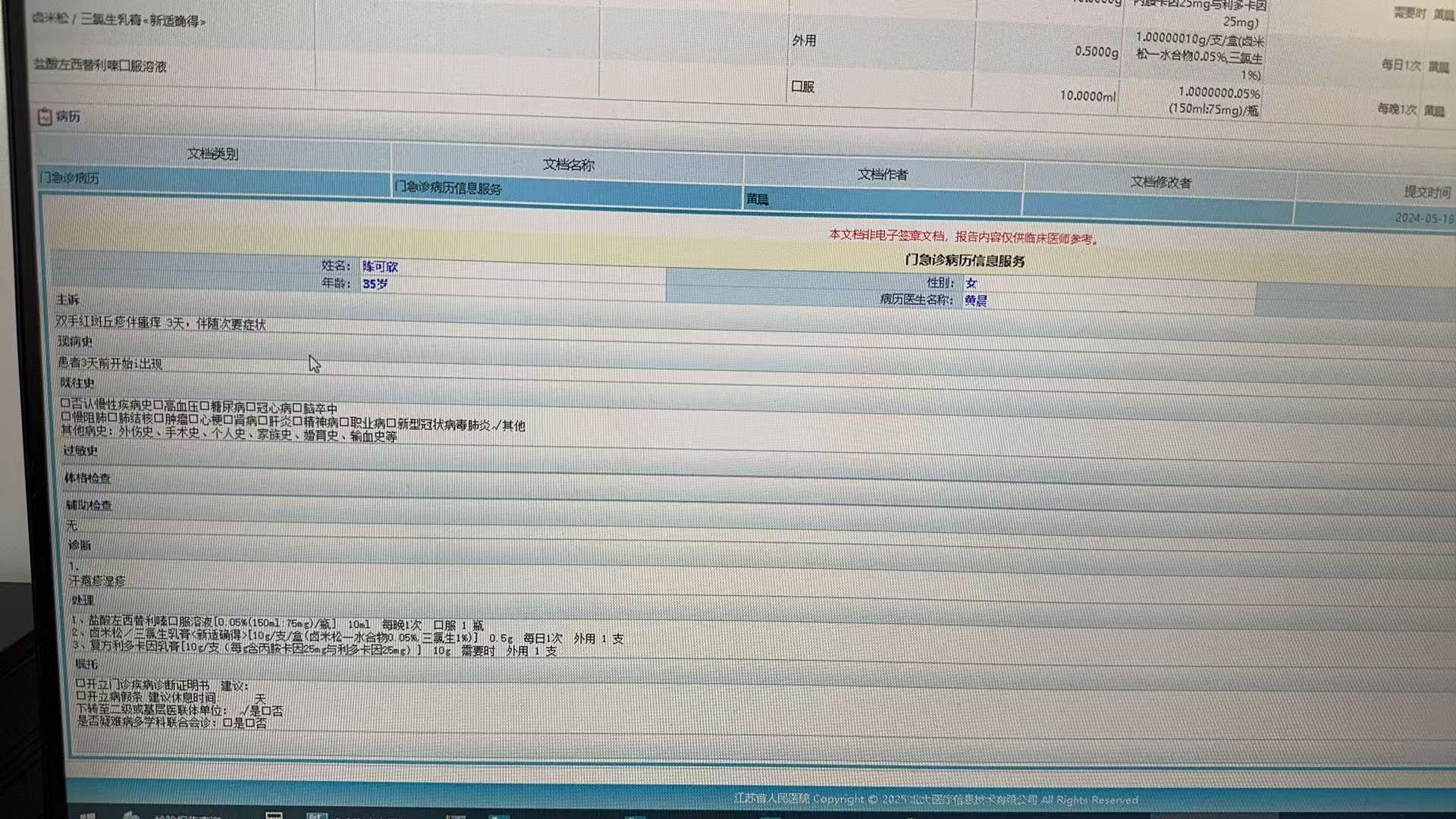


Figure 9 Dermatology medical records (2)

Figure 8 and Figure 9 showed that the patient had acute erythematous papules with itching on both hands. The dermatology department first used 5mg of dexamethasone sodium phosphate injection for emergency intravenous treatment, and then prescribed levocetirizine hydrochloride oral solution, triclosan cream and compound lidocaine cream for treatment. The patient basically recovered within a week.


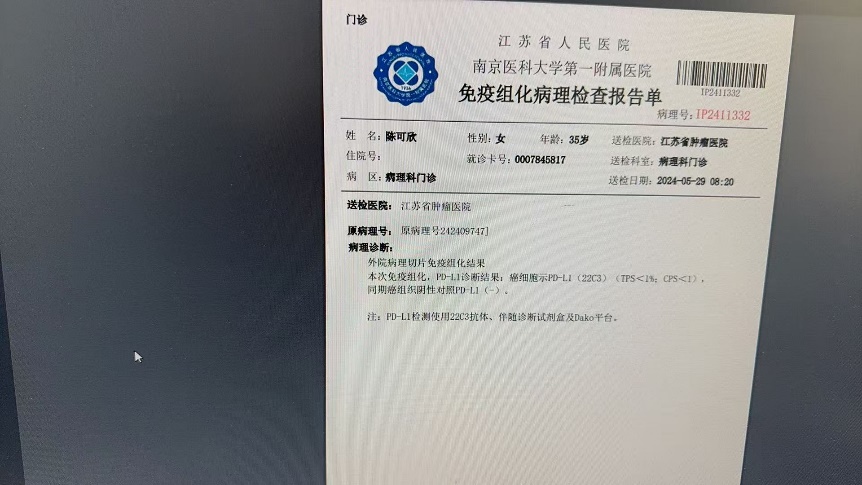


Figure 10 Ovarian tumor PD-L1 detection report

Immunohistochemistry of the oophorectomized tumor showed that PD-L1 was negative.
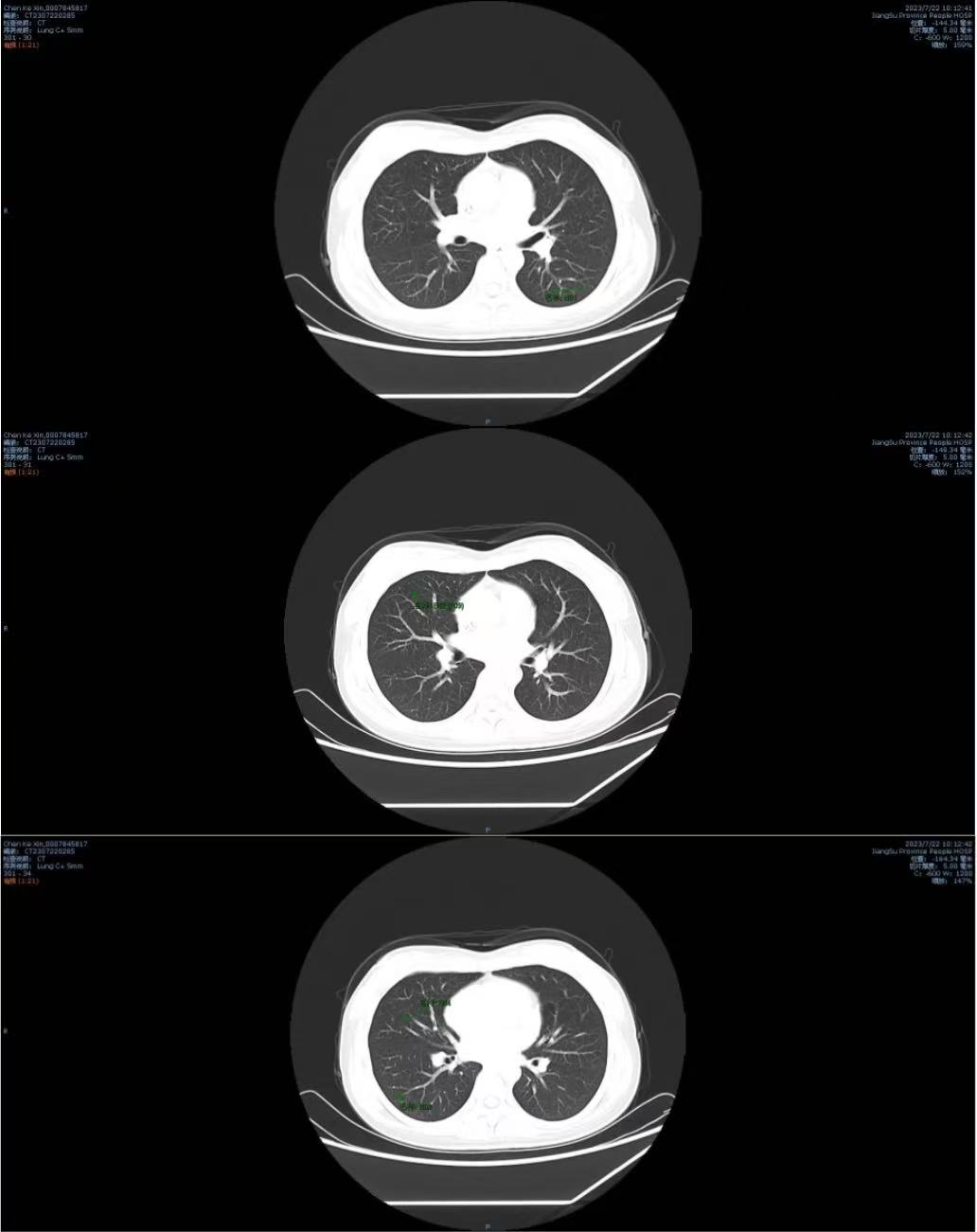


Figure 11 Enhanced CT lung metastases before starting LM302 treatment (July 24, 2023)


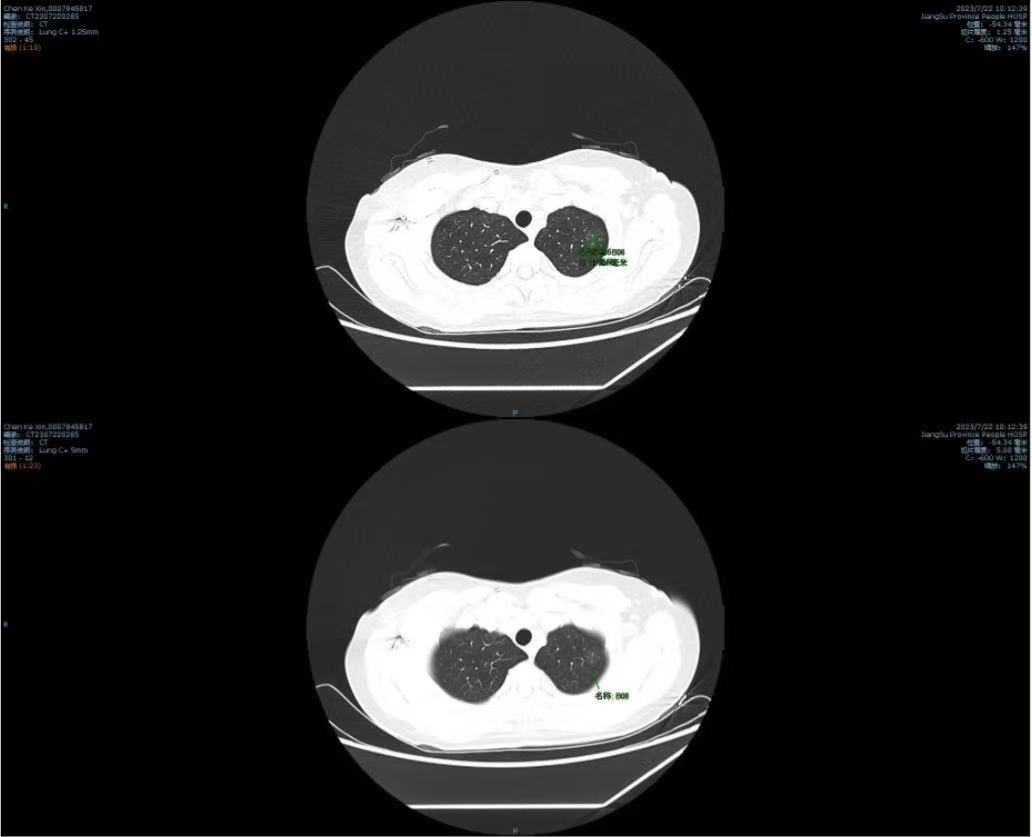


Figure 12 Enhanced CT lung metastases before starting LM302 treatment (July 24, 2023)


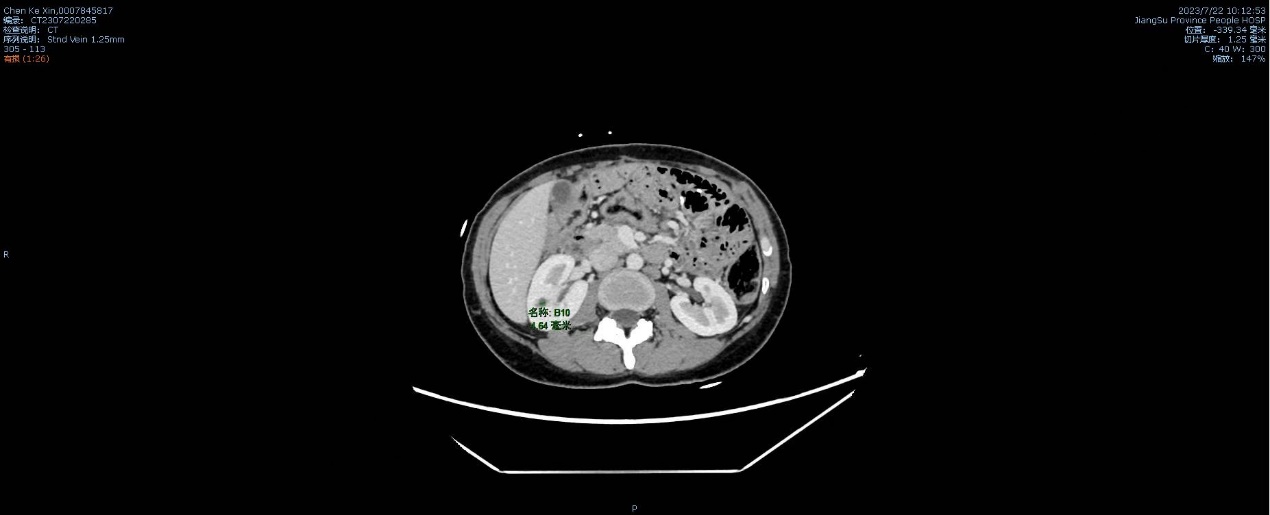


Figure 13 Enhanced CT of abdominal lymph nodes before starting LM302 treatment (July 24, 2023)


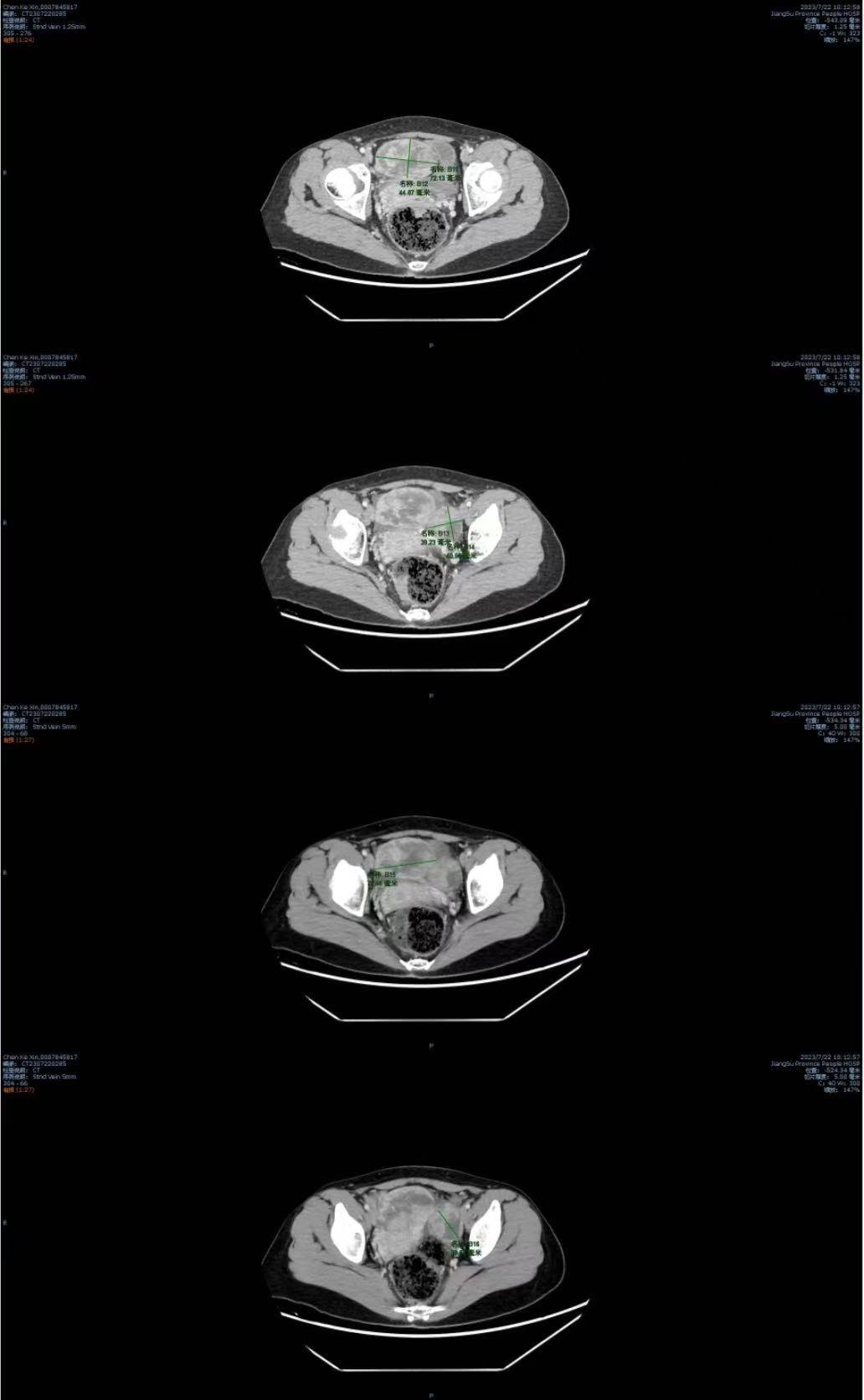


Figure 14 Enhanced CT of ovarian metastases before starting LM302 treatment (July 24, 2023)


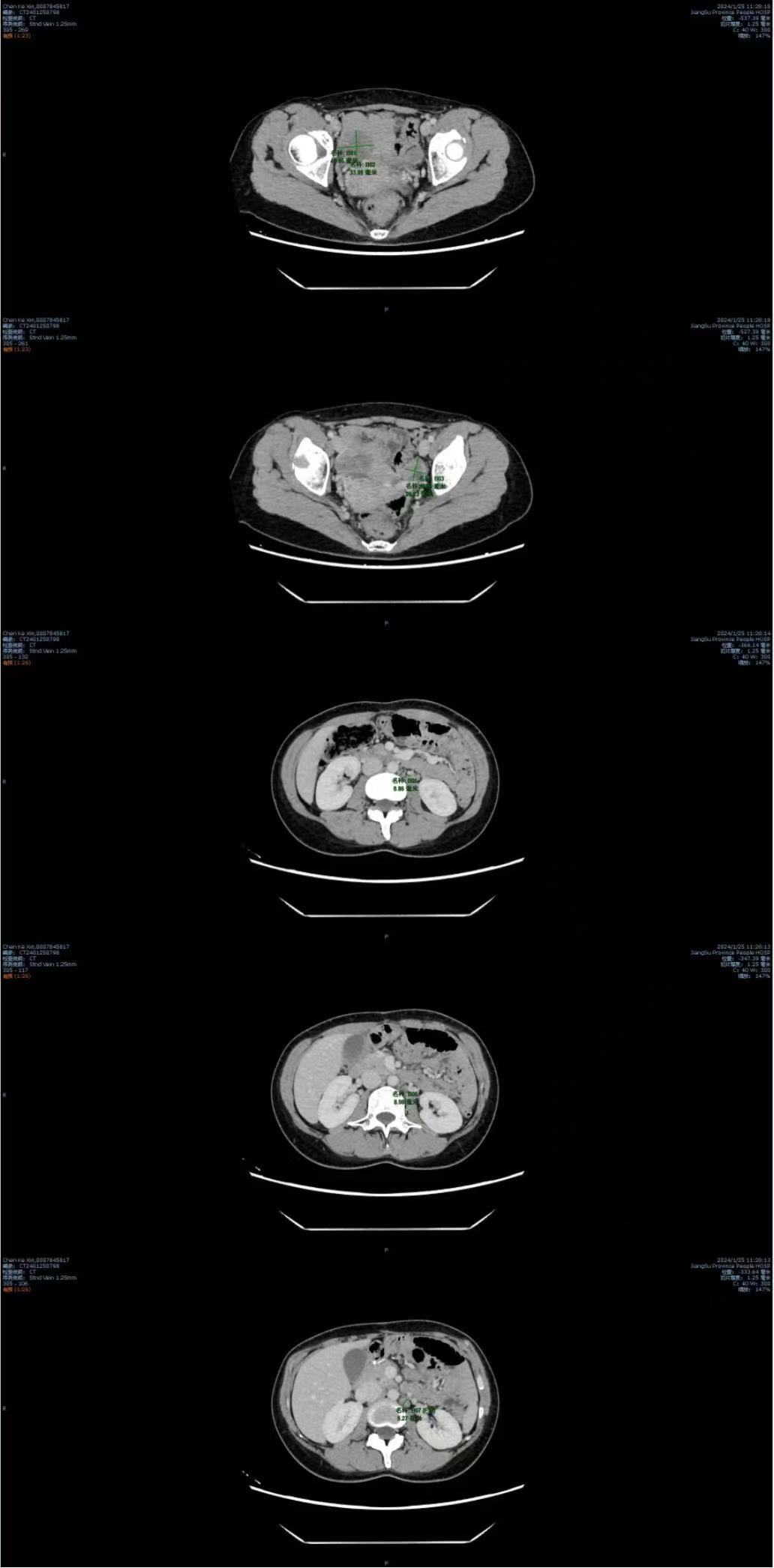


Figure 15 Enhanced CT of ovarian metastases (previous two) and abdominal lymph nodes (next three) after starting LM302 treatment (2024.1.25)


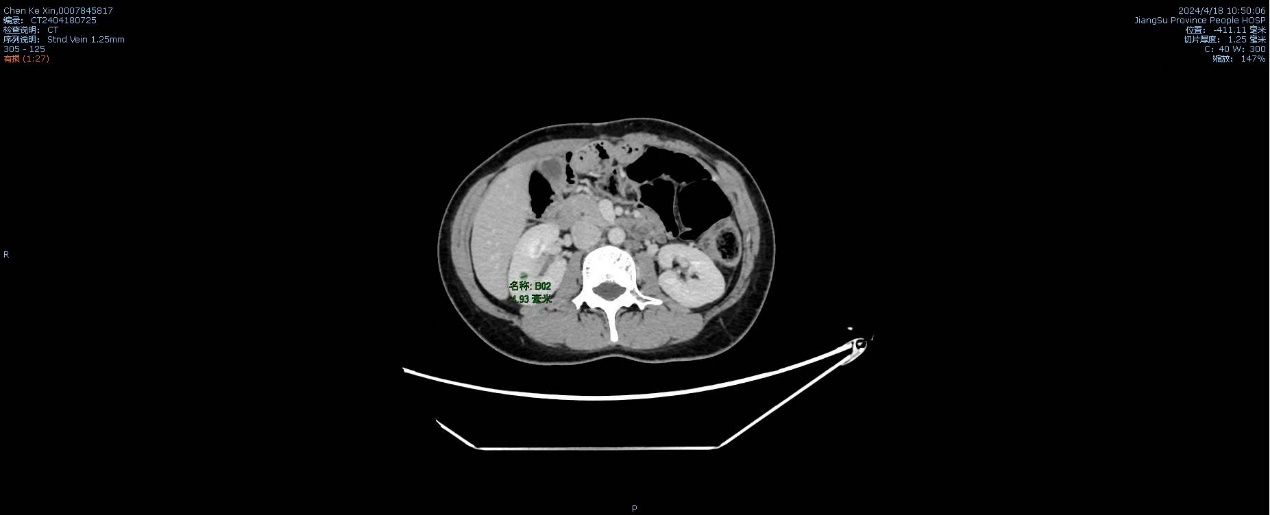


Figure 16 Enhanced CT of abdominal lymph nodes after starting LM302 treatment (April 18, 2024), similar to July 24, 2023


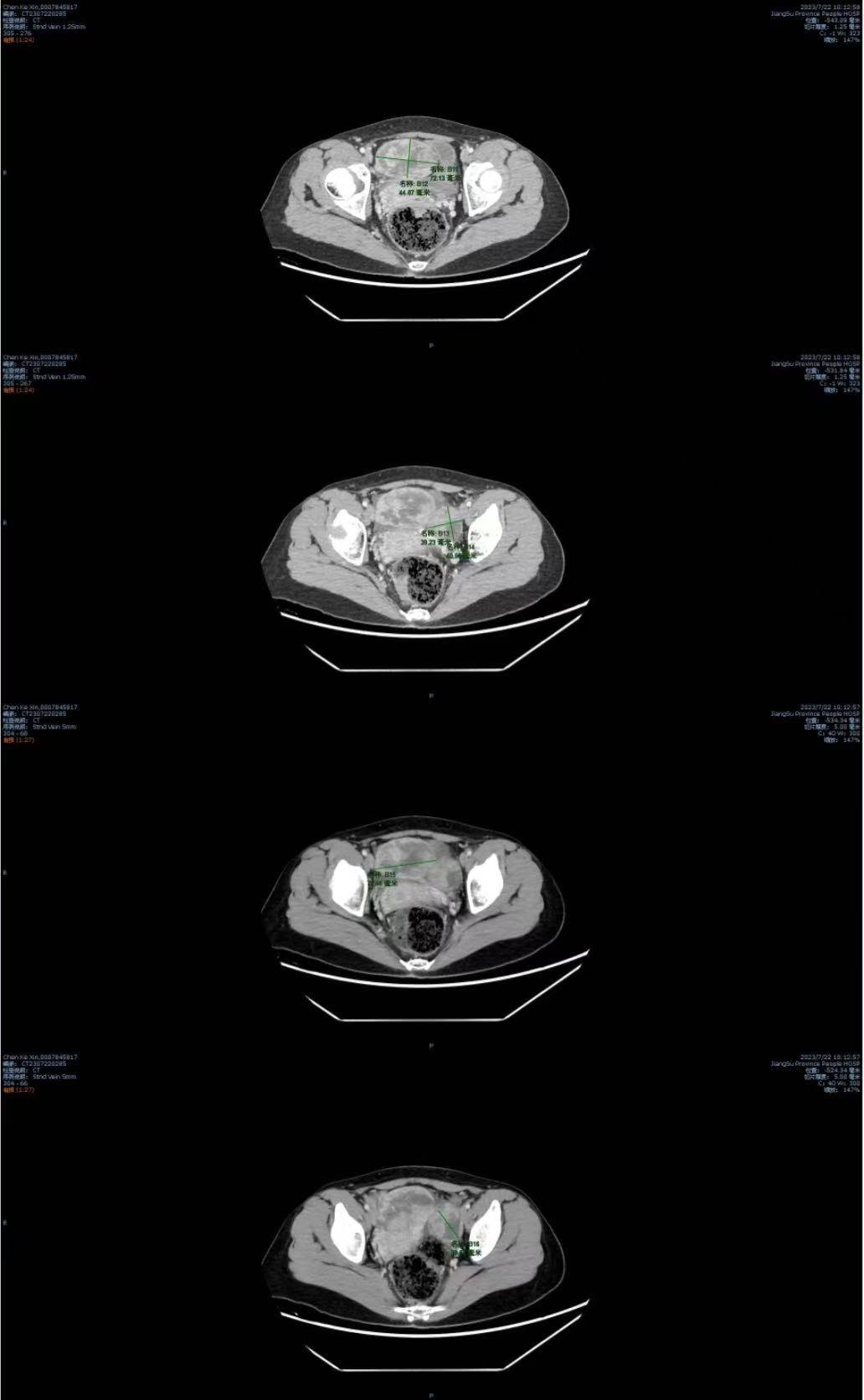


Figure 17 Enhanced CT of ovarian metastases after starting LM302 treatment (April 18, 2024). The patient underwent bilateral oophorectomy shortly thereafter.
